# Human Placental Extract Ameliorates Structural Lung Changes Iinduced by Amiodarone in Rats

**Published:** 2016

**Authors:** Fatemeh Samiei, Akram Jamshidzadeh, Ali Noorafshan, Amir Ghaderi

**Affiliations:** a *Department of Pharmacology and Toxicology, Faculty of Pharmacy, Shiraz University of Medical Sciences, Shiraz, Fars, Iran.*; b *Pharmaceutical Sciences Research Center, Shiraz University of Medical Sciences, Shiraz, Fars, Iran. *; c *Histomorphometry and Stereology Research Center, Shiraz University of Medical Sciences, Shiraz, Fars, Iran.*; d *Department of Pharmacology and Toxicology, Faculty of Pharmacy, Mashhad University of Medical Sciences, Mashhad, Iran.*

**Keywords:** Placenta, Lung, *Amiodarone*, Stereology, Rat

## Abstract

Amiodarone is used in treatment of cardiac arrhythmias. Therapeutic use of amiodarone is limited by its side effects, including pulmonary toxicity. Human Placenta Extract (HPE) contains a variety of bio-active substances. Thus, the present study aimed to quantitatively evaluate the protective effects of HPE on the structural lung changes induced by amiodarone using stereological methods. Sprague-Dawley male rats were divided into four groups. The first, second, and third groups received no treatment, amiodarone (100 mg/kg, i.p.), and HPE (500 µL/kg, i.p.), respectively. The fourth group was treated with amiodarone + HPE. The animals' lungs were removed after 10 days. The lung volume was estimated using the Cavalieri principle on the embedded and cut tissue and corrected for shrinkage. The volume density of the parenchyma, alveolar space, and septa were estimated using point-counting method. The surface area of the alveoli, the volume-weighted means alveoli volume, and mean septum thickness were also estimated in all groups. The total volume and thickness of the alveolar septum were increased by 40 % and 28 %, respectively. However, the total volume of the alveolar space was decreased by 31 % in the amiodarone treated-rats. The mean alveolar volume was decreased by 64 % on the average in the amiodarone treated group. Yet, these changes were not detected in the amiodarone+HPE group. Moreover, RBC accumulation in the alveolar space and septa was ameliorated after HPE treatment. HPE can protect the lung tissue from the structural changes induced by amiodarone.

## Introduction

Amiodarone [2-n-butyl-3-(3,5-diiodo-4-diethylaminoethoxy-benzoyl)-benzofuran] is a multiple ion (Ca^++^, Na^+^, K^+^) channel blocker drug. It is also an antiarrhythmic drug used in treatment of a wide range of cardiac arrhythmias (1). Therapeutic use of amiodarone is limited by its side effects, including pulmonary, ocular, thyroidal, and liver toxicity (2). Lung toxicity induced by amiodarone is characterized in part by phospholipidosis, inﬂammation, and thickening of the alveolar septa, intraalveolar inﬂammation, and pulmonary ﬁbrosis (3). Free radicals (oxidative stress) and lipid peroxidation have been shown to be the main pathogenic mechanisms of this drug's pulmonary toxicity (4). Diffuse alveolar damage is a common manifestation of drug-induced lung injury. Histopathologically, diffuse alveolar damage is divided into an acute exudative phase and a late reparative or proliferative phase. The exudative phase, which is characterized by alveolar and interstitial oedema and hyaline membranes, is most prominent in the 1^st^ week after lung injury. On the other hand, the reparative phase, which is characterized by proliferation of type II pneumocytes and interstitial fibrosis, typically occurs after 1 or 2 weeks. 

The placenta is usually rejected after delivery. During pregnancy, however, it is an important channel between mother and fetus providing various nutrients (4, 5). The nutritional substances and vitamins can be extracted from the placenta and are known as “placenta extract” (5). Human Placenta Extract (HPE) has been a historical folk remedy in Asian countries. In Asia specially Korea and Japan, the hydrolysate of HPE which contains therapeutic compounds has been used for improvement of liver function, liver regeneration, menopausal symptoms, fatigue, skin whitening, and antiaging (4-6). In general, placenta includes a rich variety of bio-active substances, including poly deoxyribonucleotides, RNA, DNA, peptides, hormones, amino acids, enzymes, cytokines, growth factors, vitamins, minerals, and trace elements (7). Growth factors, such as hepatocyte growth factor, epidermal growth factor, and transforming growth factor-α,-β, have been identified in HPE (5). HPE, was also reported to exhibit anti-oxidant or anti-inflammatory properties in various biological systems. HPE significantly decreased benzo[α]pyrene -induced oxidative stress which was evaluated by measuring the levels of superoxide dismutase and lipid peroxidation (4-9). In these studies, HPE pre-treatment significantly decreased the levels of the cytokines in rats exposed to benzo[α]pyrene (4-9).There are many application of HPE on tissue regeneration such as liver or wound healing but the HPE usage after lung tissue injury received less attention (6-9). The present study aims to qualitatively evaluate the protective effects of the HPE on the lung structural changes induced by amiodarone using modern stereological methods. In fact, this research was conducted to answer these questions: How much do the lung volume, alveolar septa, and space change after amiodarone treatment? How much does the surface area of the alveoli and septal thickness change after amiodarone treatment? How much does the mean alveolar volume change? Can HPE protect the lung from the structural changes induced by amiodarone? 

## Experimental

*Placenta isolation and extraction*

In this study, HPE was obtained from healthy women who had undergone cesarean section after getting verbal consent (because the placentas were discarded). Then, the placentas were processed immediately. The research procedure was approved (approval number: No. 91-6073) by the Ethics Committee of Shiraz University of Medical Sciences, Shiraz, Iran. HPE was prepared with water-soluble methods as follows. Red blood cells were removed from the placental tissue using washing buffer (50 mM Tris-HCl; 150 mM NaCl; 150 mM Sucrose, pH 7.2 each at 4 °C for 30 min). The washed tissue was then chopped using sterile scissors. Afterwards, the tissue was snap-frozen in liquid nitrogen for additional grinding, thawed, resuspended in 30 mL of homogenizing buffer (DMEM high glucose; 0.32 M sucrose; 2 mM EDTA), and sonicated on ice (amplitude 60, pulse 4, and repeat 4). After centrifugation (4500 rpm at 4 °C for 15 min), HPE was filtered by 0.45-μm filter. The protein concentration in the filtrate was determined using Bradford method and 1 mL HPE contained 50 mg protein. HPE was stored at -80 °C until use (4, 8 and 9). 

*Animals*

24 Sprague-Dawley male rats (weight, 180-220 g) were obtained from the laboratory animal center of Shiraz University of Medical Sciences, Shiraz, Iran. All the procedures were performed under the supervision of the Ethics Committee of Shiraz University of Medical Sciences, Shiraz, Iran and the animals were kept under standard conditions. The Ethics Committee of the university agreed with the animal experiment under approval No. 91-6073. The animals were randomly divided into four groups each including six rats. The first group received no treatment. The second group received amiodarone (100 mg/kg) for 10 days (10). Besides, the third group received HPE (500 µl/kg) for 10 consecutive days. The forth group was treated with the same doses of amiodarone and HPE as mentioned for the second and third groups. All the administrations were done intraperitoneally for 10 days. The animals were sacrificed on the 11^th^ day and their lungs were removed. 

The animals were anaesthetized by intraperitoneal injection of 60 mg/kg thiopental. A cannula was inserted into the trachea through a midline cervical incision while the animal was breathing. The diaphragm was punctured from its abdominal surface to produce a bilateral pneumothorax. After that, the left bronchus was ligated and the left lung was removed. Then, 4 % phosphate-buffered formaldehyde was infused into the trachea at a transpulmonary pressure of 20 cm H_2_O. The trachea was then ligated when the flow ceased (11). Afterwards, the right lung was removed from the thorax and placed in fresh fixative to complete fixation while intrapulmonary pressure was still maintained. The left lung, on the other hand, was cut into isotropic uniform random sections according to the oroientator method (12-14). Briefly, the left lung was sectioned according to an equidistance division circle. The pieces were cut using a sine-weighted non-equidistance division circle. After all, four circular pieces were sampled using a trocar and the areas were measured. The pieces were embedded in paraffin blocks, sectioned, stained, and their areas were measured to calculate the mean degree of global tissue shrinkage “d (shr)” using the following formula: 

d (shr):= 1-[Area After/Area Before]^1.5^

Where, "Area After" and "Area Before" were respectively the areas of the circle after and before fixation, processing, sectioning, and staining (13). In this study, the right lung was embedded in an agar block and systematically cut into slices starting at a uniform random point. A slicer machine was used to choose a slice thickness of 3 mm, providing about 8-12 slices from each lung. New sub-samples were also obtained by punching the slabs using a trocar through systematic random sampling method. Afterwards, the pieces were blocked in a cylindrical paraffin block and the orientator method was applied on the cylinder. Finally, 4µm isotropic uniform random sections were obtained and stained with Heidenhain's AZAN trichrome stain.

*Lung volume estimation*

**Figure 1:**
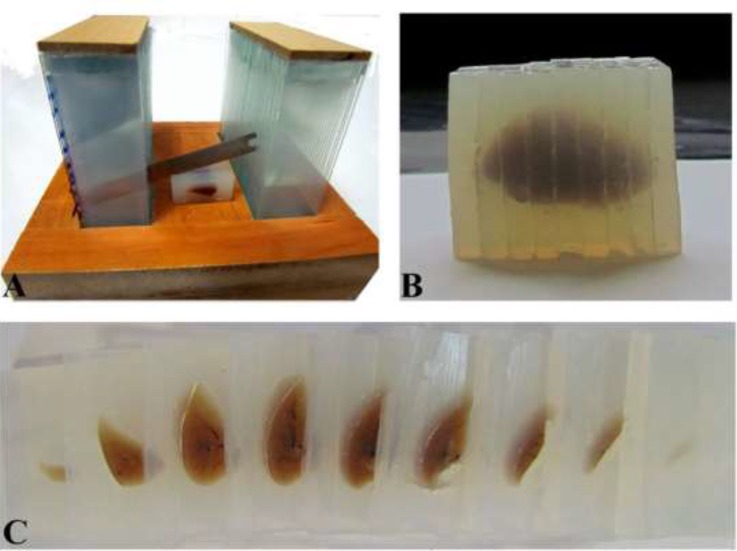
The lung sectioning. A. The lung was embedded in an agar block and cut using a tissue slicer. B and C. The sectioned block and slabs of the lung

The lung volume was estimated using the Cavalieri principle on the embedded and cut tissue (Figure 1). Also, it had to be corrected for shrinkage to achieve values for unshrunken lung volumes (11). The images of the slabs were captured using a stereomicroscope and the areas of the slabs were estimated. The lung volume was estimated using the following formula: 

V (lung, shrunken)= ∑A×T

Where "∑A" was the areas of the slabs and "T" was the slab thickness. Moreover, the unshrunken volume of the lung was estimated using the following formula.

V(lung, unshrunken)= V(lung, shrunken) /[1 -d(shr)]

*Estimation of the Coefficient of Error* (*CE*)

The CE for the estimate of the volume; i.e, CE (V), is the function of the noise effect and systematic random sampling variance for the sums of areas. When the cross-sectional areas "∑A" were estimated by the software, CE (V) was calculated by the following formula (15^,^^16^):

CE(V):= (∑A)^-1^ × [1/12 ×(3∑A_i_A_i_ +∑A_i_A_i+2_ - 4∑ A_i_A_i+1_)]^1/2^

*Volume density of the lung tissue*

**Figure 2:**
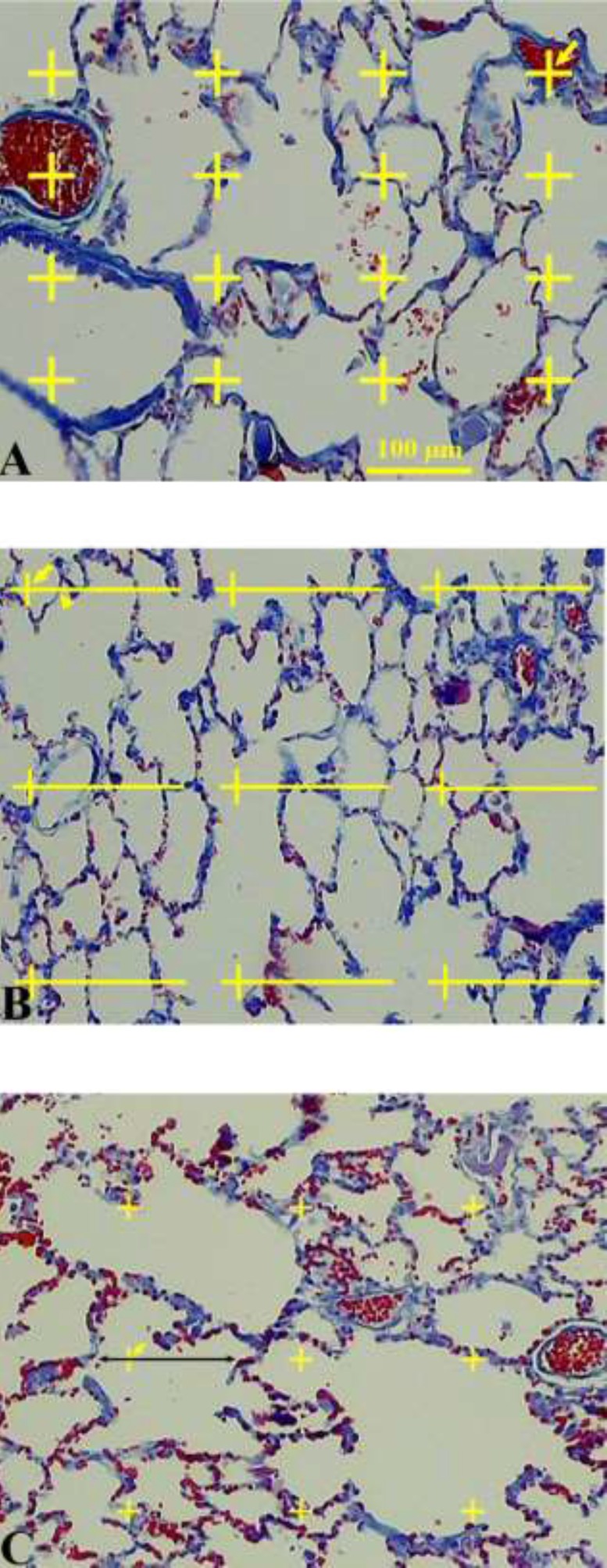
A. Volume density estimation using point counting method. The number of points hitting the favored structure was divided by the total points hitting the lung tissue. The right upper corner of the crosses indicates the points (the arrows). B. Surface density estimation. The points and intersections are shown using arrows and arrowheads, respectively. C. Estimation of the volume-weighted mean alveolus volume. Each alveolus hit by a test point was sampled and the intercept length was calculated

The data about the changes in the distribution of the volume fractions of the lung tissue on different subtypes of the tissue were collected using the point counting method (Figure 2) (11-13). Briefly, a grid of points was superimposed upon the images of the lung sections viewed on the monitor. The right upper corner of each cross point was considered as a point. In the first step, the points hitting the parenchymal tissue and those hitting the non-parenchymal tissue were calculated at the final magnification of 40×. The parenchymal tissue includes the part of the lung tissue that belongs to alveolar septa and spaces. The non-parenchymal tissue was defined as the large connective tissue septa, walls of the larger vessels, and conductive airways and their surrounding connective tissue. Overall, 100-278 points were counted per animal. In the second step, the volume density of the alveolar septa and alveolar space was estimated at the final magnification of 400×. The volume density “Vv (structure/lung)” of the intended parameters was obtained using the following formula:

Vv (structure/lung):=P (structure) / P(lung)

Where "P (structure)" and "P (lung)" represented the total number of the points hitting the structural components of the tissue and the lung sections, respectively. Overall, 128-241 points were counted per animal. The total volume of the *parenchymal *tissues was estimated by multiplying the volume density by V (lung, unshrunken) (11). 

V (parenchyma)= Vv (parenchymal/lung) × V(lung, unshrunken)

The total volume of the alveolar septa and space were estimated using the following formula: 

V (structure)= Vv (structure/parenchyma) × V (parenchyma)

In order to estimate the surface area of the alveoli, S (alveoli), the intersections of the alveolar boundaries on the section with straight test lines of a superimposed grid were counted at the final magnification of 400× (Figure 2) (11). The alveolar surface area on the sections was estimated as follows: S (alveoli)= 2∑I / [(*l/p*)×∑ P(lung)] × [1 -d(shr)]^1/3^ × V (parenchyma).

Where "∑I" was the number of the intersections between the test lines and the alveolar septa, "*l/p*" was the length per test point on the counting grid, and P lung) was the number of the points hitting the lung tissue. Overall, 226-678 intersections were counted per animal.

*Volume-weighted mean alveoli volume*

The volume weighted mean volume (*V*v) of the alveoli was estimated by the method of point sampled intercepts (Figure 2) (17). This method is based on the following formula:

$Vv=\pi /3\times \overset{\text{̅}}{l_{0}^{3}}$

Where "*l*_0_" represented intercept. In brief, a test grid was overlaid on the section image of the lung at the final magnification of 400×. Each section hit by a test point was sampled and the intercept length was calculated. Due to the random orientation of the alveoli, the ruler was maintained in a horizontal position to determine the intercepts. Overall, 88-267 intercepts were estimated per animal.

*Mean thickness of the alveolar septum*

The mean septum thickness "τ" was estimated as:

τ =2×(Vv septum /Sv septum)

Where "Vv" and "Sv" were the volume and surface densities of the alveolar septum, respectively (18).

*Statistical analysis. *The data were analyzed using Kruskall-Wallis and Mann-Withney U-tests. P<0.05 was considered as significant. 

## Results

The quantitative data are presented in Table 1. No changes were observed in the total lung and parenchyma volume 10 days after amiodarone treatment.

On the average, the total volume of the alveolar septum increased by 40% in the amiodarone treated rats. However, this increase was not found in the group which was treated by amiodarone+HPE.

Moreover, the total alveolar space was averagely decreased by 31% in the amiodarone treated animals. Nonetheless, this reduction was not observed in the amiodarone+HPE group. 

Furthermore, the thickness of the alveolar septum was averagely increased by 28% in the amiodarone-treated group. Yet, this increase in thickness was not detected in the rats treated with amiodarone+HPE. 

The study results also showed that the mean alveolar volume was decreased by 64% on the average in the amiodarone-treated group. However, this reduction was not found in the amiodarone+HPE group. 

**Figure 3:**
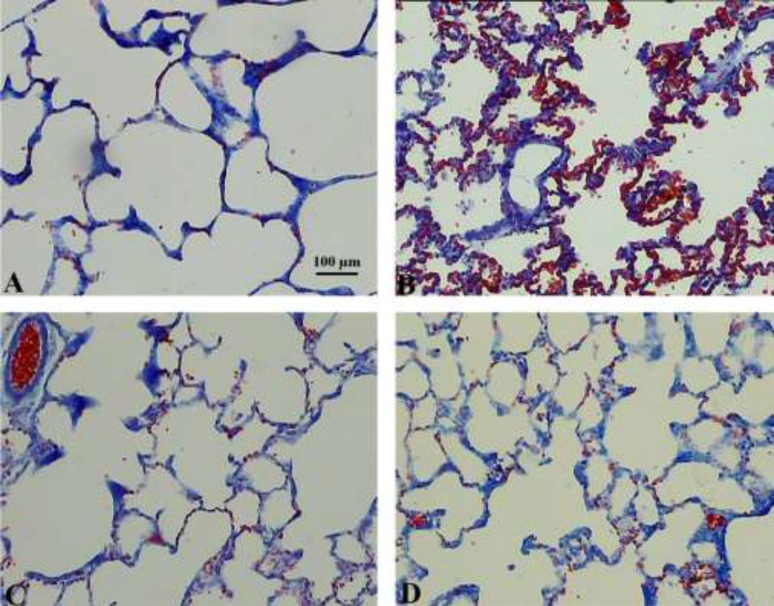
Hisotopathological observation of the lung tissue in the control (A), amiodarone (B), HPE (C), and amiodarone+HPE (D) groups. Diffuse alveolar damage can be seen in amiodarone-treated lungs characterized by alveolar and interstitial oedema and *RBC* accumulation in the *alveolar space. No changes were seen after HPE-treatment on the normal lung tissue. *These histopathological characteristics of the lung injury were decreased in the amiodarone+HPE extract lungs

Qualitative changes of the lung tissue are presented in Figure 3. RBC accumulation in the alveolar space and septa and septal inflammation were observable in the amiodarone-treated rats. In addition, the amelioration of these characteristics could be seen after HPE treatment. 

## Discussion

Recent research has shown that HPE contains rich growth factors, hormones, proteins, glycosaminoglycans, nucleic acids, polydeoxyribonucleotides, antibodies, and other concentrated nutrients which may explain in part the capacity of HPE to stimulate tissue regeneration and cell proliferation (4-9). Moreover, uracil, tyrosine, phenylalanine, tryptophan, and collagen peptides are responsible for the antioxidant activity of HPE (9). Since HPE has been known to have no toxic effects, it can be used as a therapeutic agent for oxidative stress-related diseases (19). The studies conducted on animal models have also provided evidence for liver function improvement by HPE through liver regeneration (20). 

Amiodarone, an antiarrhythmic drug, causes pulmonary fibrosis in some patients during chronic administration, but its toxic mechanism is not well known. Formations of free radicals and lipid peroxidation (oxidative stress) have been shown to be the main pathogenic mechanisms of this drug's pulmonary toxicity (21). The present study investigated the protective effects of HPE on amiodarone-induced lung injury in rats in a quantitative way. 

The study was focused on the structural changes after amiodarone with or without HPE treatments. The study results showed that thickness and total volume of the alveolar septum increased after amiodarone administration and HPE could suppress these changes. In addition, our findings revealed a decrease in the mean alveolar volume and total alveolar space which are important characteristics of amiodarone lung toxicity. Other researchers have proposed an oxidant mechanism for amiodarone and have shown that several antioxidants can reduce lysosomal phospholipidosis and prevent amiodarone toxicity (4, 22). 

The Exact mechanism of the HPE action is not clear but it might be due to its antioxidant properties. In general, HPE contains many kinds of collagens, including type I, II, III, IV, V, VII, VIII, and XVI (23). Reactive oxygen species inhibit collagen synthesis in a variety of tissues, including lung, skin, and venous endothelial cells (9). Due to the anti-inflammatory properties and collagen contents, HPE might be improved amiodarone-induced inflammation and thickening of the alveolar septa within the lung in the current study. The protective effects of HPE against amiodarone observed in the present study might also be exerted by the components of HPE, including amino acids, monosaccharides, and fatty acids. It should also be considered that the cell-regenerative effect of HPE may stimulates tissue regeneration and cell proliferation in the lung tissue. 

Conclusion. HPE may protect the lung tissue from the structural changes induced by amiodarone in rat. 
